# Widespread distribution of *mcr-1-*bearing bacteria in the ecosystem, 2015 to 2016

**DOI:** 10.2807/1560-7917.ES.2017.22.39.17-00206

**Published:** 2017-09-28

**Authors:** Kaichao Chen, Edward Wai-Chi Chan, Miaomiao Xie, Liangwei Ye, Ning Dong, Sheng Chen

**Affiliations:** 1 Shenzhen Key lab for Food Biological Safety Control, Food Safety and Technology Research Center, Hong Kong PolyU Shen Zhen Research Institute, Shenzhen, P. R. China; 2 State Key Lab of Chirosciences, Department of Applied Biology and Chemical Technology, The Hong Kong Polytechnic University, Hung Hom, Kowloon, Hong Kong; 3 These authors contributed equally to the work

**Keywords:** *mcr-1*, Enterobacteriaceae, isolation method, food, water, animal, human, ecosystem, distribution

## Abstract

The recently discovered colistin resistance-encoding element, *mcr-1*, adds to the list of mobile resistance genes whose products rapidly erode the antimicrobial efficacy of not only the commonly used antibiotics, but also the last line agents of carbapenems and colistin. The relative prevalence of *mcr-1*-bearing strains in various ecological niches including 1,371 food samples, 480 animal faecal samples, 150 human faecal samples and 34 water samples was surveyed using a novel in-house method. Bacteria bearing *mcr-1* were commonly detected in water (71% of samples), animal faeces (51%), food products (36%), and exhibited stable carriage in 28% of human subjects surveyed. Such strains, which exhibited variable antibiotic susceptibility profiles, belonged to various Enterobacteriaceae species, with *Escherichia coli* being the most dominant in each specimen type. The *mcr-1 *gene was detectable in the chromosome as well as plasmids of various sizes. Among these, two conjugative plasmids of sizes ca 33 and ca 60 kb were found to be the key vectors that mediated *mcr-1* transmission in organisms residing in various ecological niches. The high *mcr-1* carriage rate in humans found in this study highlights the importance of continued vigilance, careful antibiotic stewardship, and the development of new antimicrobials.

## Introduction

The effectiveness of antibiotics to combat bacterial infections has diminished rapidly in the past decade due to incessant emergence of bacterial strains that exhibit novel and transmissible resistance mechanisms, such as carbapenem-resistant Enterobacteriaceae (CRE) strains that commonly cause untreatable and hard-to-treat infections among hospitalised patients. CRE are now considered an urgent public health threat according to reports by the European Centre for Disease Prevention and Control (ECDC) and the United States (US) Centers for Disease Control and Prevention (CDC) [1,2].

Colistin is currently considered a last-resort antibiotic that can be used to treat clinical CRE infections. Bacterial resistance to colistin was previously thought to be rare, and mainly attributed to chromosomal mutations leading to modification of lipid A or loss of lipopolysaccharide [3,4]. Recently, a new plasmid-encoded colistin resistance mechanism, mediated by the MCR-1 protein, a phosphoethanolamine transferase that modifies the phosphoethanolamine moiety of lipid A, has been reported [5]. Since its discovery in November 2015, the *mcr-1* genehas been associated with a wide range of mobile elements in different bacterial species, suggesting that this resistance element is highly transmissible, thereby posing a huge challenge to the use of colistin as a reserved drug for treatment of CRE infections [6]. Currently there is a lack of studies comprehensively screening for *mcr-1* positive bacteria in the environment, and the most frequent sources investigated for such bacteria consist of human clinical samples and veterinary specimens. Hence, information on the prevalence of *mcr-1* gene in various ecological niches is not available to assess of the degree of *mcr-1* contamination, which can potentially impact the clinical use of colistin. This is due to a lack of methods for specific isolation of *mcr-1*-positive bacteria, since many species of bacteria are intrinsically resistant to colistin, interfering with the isolation of *mcr-1*-positive organisms. In this study, we have developed a novel method that facilitates specific detection of *mcr-1*-positive bacteria. Using this method, we checked various environmental sources in China for the presence of *mcr-1* positive bacteria. These included animal and human faecal samples, as well as sewage water, seawater and fresh water samples. Foods locally produced or imported from overseas were also tested.**

## Methods

### Specific isolation of *mcr-1*-bearing bacteria

#### PCR screening for *mcr-1*-bearing organisms in food specimens

A PCR-based method was first developed to screen for the presence of *mcr-1*-bearing organisms in food products. A total of 25 g of each food sample was mixed with 75 mL alkaline peptone water and stomached for 30 s; 50 mL of the suspension (without sediment) were then incubated in a tube overnight at 37 °C without shaking. After incubation, the tube was inverted several times and left standing for 10 min for settlement of the food particles. To collect the bacteria, 1 mL of the suspension was centrifuged at 13,000 rpm, and the pellet was subsequently washed twice with phosphate buffered saline (PBS). The final pellet was resuspended in 100 µL of saline water and boiled at 100 °C for 5 min to release bacterial DNA. The sample was then subjected to centrifugation at 13,000 rpm for 10 min. The supernatant was then used as DNA template for PCR with the primers targeting *mcr-1* as reported previously [5]. The genetic identity of all amplification products was confirmed by nt sequencing.

#### Isolation of Enterobacteriaceae containing *mrc-1* in food

The samples that were positive for the *mcr-1* gene were subjected to isolation of *mcr-1*-positive Enterobacteriaceae strains using the following procedure. Briefly, overnight-incubated food sample suspension as mentioned above was inverted several times to resuspend the bacteria, and then left standing for 10 min to facilitate settlement of large particles. 500 µL enrichment broth at the top of the tube were then removed and added into 5 mL Mossel Enterobacteria enrichment broth (MEE broth, Sigma-Aldrich) supplemented with 0, 2, 4, 8, 16, 32 and 64 µg/mL colistin E, followed by incubation at 37 °C overnight. A standard loop of overnight MEE culture was inoculated onto MacConkey agar plate and incubated at 37 °C overnight. Twenty colonies with different morphologies were selected for further purification and confirmation of the presence of *mcr-1* by PCR as previously described [5]. The optimal concentration of colistin used in MEE broth, which could specifically select for *mcr-1*-bearing organisms, was determined. The *mcr-1* negative samples as defined by the PCR assay were also subjected to isolation of *mcr-1*-bearing bacteria as described above.

#### Limit of PCR detection and isolation of *mcr-1*-bearing organisms in food samples

To determine the limit of detection and isolation of *mcr-1*-bearing bacteria for the above methods, three *mcr-1* negative samples were selected for the following assays. 9.9 mL of pork suspension prepared as described above was inoculated with 10°, 10^1^, 10^2^, 10^3^, 10^4^ and 10^5^ CFU of *mcr-1*-positive *Escherichia coli* isolates in 100 µL volume. Two *E. coli* isolates, SZM584, carrying a plasmid-borne *mcr-1* gene, and SZM485, carrying the *mcr-1* gene in the chromosome, were used. After overnight incubation at 37 °C, PCR template was prepared and subjected to PCR detection as described above. Similarly, pork suspensions inoculated with various amounts of *mcr-1*-positive *E. coli*, were subjected to isolation of *mcr-1*-bearing bacteria as described above, using an optimal concentration of colistin as determined above.

#### Isolation of *mcr-1*-bearing organisms from water and sewage

For isolation of *mcr-1*-positive Enterobacteriaceae strains from water and sewage, samples were collected at each site and filtered by vacuum filtration using a filter membrane (< 0.2 μm). Bacteria on the membrane was then suspended in 10 mL sterile 0.45% saline solution. 200 μL of the suspension were inoculated into 10 mL alkaline peptone water and incubated at 37 °C overnight. Subsequently, 500 µL of the later mixture were added to 5 mL MEE broth containing 2 µg/mL colistin E and incubated at 37 °C overnight. Bacterial isolation was then performed as described above.

#### Isolation of *mcr-1*-bearing organisms from faecal samples

For faecal samples, 5 g each were suspended in 15 mL alkaline peptone water and stomached for 30 s; 10 mL of the suspension were incubated overnight at 37 °C without shaking. After incubation, the enrichment broth was mixed and allowed to settle for 10 min. 500 µL of enrichment broth containing a positive sample was added into 5 mL MEE broth containing 2 µg/mL colistin E and incubated at 37 °C. The isolation procedure was the same as described above.

### Surveillance of *mcr-1*-positive Enterobacteriaceae recovered from various niches in the ecosystem

Food samples including meat products (chicken, duck, pork, beef and mutton), seafood (shrimp, fish and shell fish), dairy products (yogurt, milk cheese and butter), fresh produce (lettuce, cabbage, broccoli, cauliflower etc.) and other food products (tofu etc.) were purchased from supermarkets and wet markets located in different districts of Shenzhen, mainland China (each of four different supermarkets and wet markets) and Hong Kong Special Administrative Region (each of three different supermarkets and wet markets). Meat and seafood products of overseas origin including Asian countries, Australia, Brazil, Canada, Denmark, New Zealand, Norway, and the US were collected from various stores of the three biggest chain supermarkets located in three districts of Hong Kong. The different districts chosen for this sampling in both Shenzhen and Hong Kong were all highly and densely populated residential areas. Water and sewage samples were collected from different bay areas, waste water treatment plants and a fresh water reservoir in Shenzhen. Human faecal samples were obtained from in- and outpatients in four different hospitals located in Shenzhen city and adjacent areas. Pet faecal samples were collected from three animal hospitals located in various geographical sites in Shenzhen city. Chicken and pig faecal samples were collected from farms located in the Chinese provinces of Fujian, Guangdong, Henan, Hubei, Jiangshu, Shandong, Shanxi and Zhejiang. All samples were collected during the period of December 2015 to May 2016. All samples were subjected to *mcr-1*-positive bacteria isolation using methods described above.

### Antimicrobial susceptibility tests and detection of beta-lactamase genes

All Enterobacteriaceae isolates were subjected to antimicrobial susceptibility tests according to the standard agar dilution method described by the Clinical and Laboratory Standards Institute (CLSI) [7,8]. Sixteen antimicrobials were tested (as presented in the table shown in the result section). *E. coli* strain ATCC 25922 was used as a control. Beta-lactamase genes in cephalosporin-resistant *E. coli* isolates were determined by PCR as previously described [9].

### Conjugation, S1-pulsed-field electrophoresis and Southern hybridisation

Conjugation experiments were carried out using the mixed broth method as previously described [10]. Pulsed-field gel electrophoresis (PFGE), S1-PFGE and Southern hybridisation were performed as previously described [11]. The chi-squared test was used to compare the conjugation rate among *E. coli* strains isolated from different sources.

### Complete plasmid sequencing

One each of representative plasmids containing *mcr-1*, one of ca 33 kb and the other ca 60 kb in size, was recovered from transconjugants for sequencing. Plasmid sequencing was performed as previously described using the Illumina and PacBio RS II platforms [6]. All plasmid sequences were submitted to Rapid Annotation using Subsystem Technology (RAST) tool for annotations and modified manually by Basic Local Alignment Search Tool (BLAST) [12]. BLAST Ring Image Generator (BRIG) software was used to compare plasmids [13]. The nt sequences of the ca 33 kb plasmid (pECJS-B65–33) and the ca 60 kb plasmid (pECJS-61–63) were submitted to GenBank; the accession numbers were KX084392 and KX084393 respectively.

## Results

### Method development

The PCR assay was able to detect the *mcr-1* gene directly from various food samples. For isolation of *mcr-1*-positive bacteria from *mcr-1* positive samples, 10 colonies with pink to red colour (suspicious for *E. coli* and *Klebsiella pneumoniae*) were picked from MacConkey plates spread with MEE broth supplemented with 2 µg/mL of colistin; all the 10 colonies were confirmed to be *mcr-1* positive. Consistently, 10 colourless to yellow colonies (indicative of non-*E. coli* strains) collected from these plates were found to be *mcr-1* negative. Eight of the 10 red colonies from MacConkey plates spread with MEE broth supplemented with 4 µg/mL of colistin were *mcr-1* positive and all 10 yellow colonies were negative for *mcr-1*. However, MEE broth supplemented with 0, 1, 8, 16 and 32 µg/mL of colistin failed to isolate any *mcr-1*-bearing bacteria. All red or yellow colonies collected from MacConkey plates spread with these MEE broth were all negative for *mcr-1*. These data suggested that MEE broth supplemented with 2 µg/mL was optimal for specific isolation of *mcr-1*-bearing bacteria from food products. Using this method, the *mcr-1* gene could be successfully detected by PCR in 9.9 mL of pork suspension inoculated with 10^3^ CFU or more of *mcr-1*-positive *E. coli* strains, SZ584 or SZ485, which was equivalent to 10^4^ CFU *mcr-1*-positive *E. coli* per 25 g of pork. For isolation of *mcr-1*-bearing organisms, *E. coli* strains SZ584 or SZ485 could be successfully isolated from 9.9 mL of pork suspension inoculated with 1×10° CFU or more of SZ584 or SZ485, which was equivalent to 10^1^ CFU *mcr-1*-positive *E. coli* per 25 g of pork. Our data suggested that the isolation method was more sensitive than the PCR detection method. Therefore, the isolation method was used in subsequent surveillance experiments.

### Prevalence of *mcr-1-*bearing bacteria in various food and environmental samples

#### Food samples

A total of 1,371 food samples obtained in Shenzhen and in Hong Kong, including 234 samples from overseas-imported foods, were subjected to screening of *mcr-1*-bearing organisms; 498 (36%) positive samples were identified.

Among the 620 food samples surveyed in Shenzhen, *mcr-1*-bearing bacteria isolates were isolated in 150 of 230 (65%) meat samples and 27 of 390 (7%) other food samples (**Table 1**). Among the 230 meat samples, the *mcr-1* isolation rate was 107/142 (75%), 29/43, 2/4, 2/6 and 10/35 for pork, chicken, duck, mutton and beef, respectively. The isolation rate in meat products purchased from supermarket (61/103, 59%) was slightly lower than that of meat purchased from wet market (89/127, 70%). In addition, seafood and vegetable products were also contaminated with *mcr-1*-bearing bacteria, with a rate of 9/63 (14%) and 18/271 (7%) respectively. All dairy products and other food such as tofu were negative for *mcr-1*-bearing bacteria.

**Table 1:** Prevalence of *mcr-1*-bearing bacteria in food, environmental, animal and human faecal samples, December 2015−May 2016

Specimen types	Number of specimens	Number of positive samples	% positive	Bacterial species recoverable (number of isolates)
**Food**	1,371	498	36	*Escherichia coli* (730), *Klebsiella pneumoniae* (3), *Aeromonas* *veronii* (4)
Shenzhen	620	177	29	
Meat	230	150	65	
Others	390	27	7	
Hong Kong	517	215	42	
Meat	376	196	52	
Others	141	19	13	
Overseas	234	106	45	
Meat	222	104	47	
Other	12	2	17	
**Animal faeces**	480	243	51	*E. coli* (576), *K. pneumoniae* (10), *Enterobacter cloacae* (4)
Pig^a^	245	124	51	
Chicken^a^	180	113	63	
Pet^b^	55	6	11	
**Human^c^**	150	42	28	*E. coli* (84), *E. cloacae* (5)
Inpatients	85	22	26	
Healthy individuals	65	20	31	
**Water**	34	24	71	*E. coli* (77), *K. pneumoniae* (4), *K. variicola* (3)
Sewage	24	18	75	*E. coli* (52), *K. pneumoniae* (4), *K. variicola* (3)
Seawater	6	6	100	*E. coli* (25)
Fresh water	4	0	0	None (0)

^a^ Chicken and pig faecal samples were collected from farms located in the Provinces of Fujian, Guangdong, Henan, Hubei, Jiangshu, Shandong, Shanxi and Zhejiang. The contamination rate of faecal samples collected from farms located in different provinces of China was 63% (25/40) in Fujian, 14% (8/58) in Guangdong, 55% (30/55) in Henan, 83% (75/90) in Hubei, 56% (28/50) in Jiangshu, 100% (42/42) in Shandong, 70% (28/40) in Shanxi, and 50% (25/50) in Zhejiang.

^b^ Pet faecal samples were collected from three animal hospitals located in various geographical sites in Shenzhen city.

^c^ Human faecal samples were obtained from in- and outpatients in four different hospitals located in Shenzhen city and adjacent areas.

The origin of food products surveyed in Hong Kong were categorised as either ‘Hong Kong’ or ‘overseas’. Among the 517 food samples tested in Hong Kong, which had not been imported from overseas, *mcr-1*-bearing bacteria isolates were isolated in 196 of 376 (52%) meat samples and 19 of 141 (13%) other food samples (**Table 1**). The *mcr-1*-bearing bacteria isolation rate in different types of food samples was similar to that of Shenzhen except that vegetable products exhibited a higher contaminated rate (12/77, 15%) of *mcr-1*-bearing bacteria than those from Shenzhen. Food of overseas origin mainly included meat products and seafood (a total of 234 samples), in which the *mcr-1*-bearing bacteria isolation rate was 104 of 222 (47%) meat samples and 2 of 12 seafood samples respectively (**Table 1**). The *mcr-1*-bearing bacteria isolation rates varied slightly between different countries in different regions of the world, with rates of 4/12, 37/69 (54%), 28/51 (55%), 27/72 (38%) and 11/18 recorded in food products originating from Asia, US/Canada, Brazil, Australia/New Zealand and Denmark/Norway respectively. Our data also indicated that meat products showed the highest contamination rate of *mcr-1*-bearing bacteria among all types of food products. Among the *mcr-1* positive food samples recovered in both Shenzhen and Hong Kong, a total of 737 bacterial strains containing the *mcr-1* element were isolated from the food samples tested, the majority of which (n = 730) being *E. coli*, followed by *Aeromonas veronii* (n = 4) and *K. pneumoniae *(n = 3)**(**Table 1**).

#### Animal faecal samples

The *mcr-1*-bearing bacteria isolates were detected in 51% (124 of 245) and 63% (113 of 180) of pig and chicken faecal samples, respectively (**Table 1**). The contamination rate of faecal samples collected from farms located in different provinces of China was 63% (25/40), 14% (8/58), 55% (30/55), 83% (75/90), 56% (28/50), 100% (42/42), 70% (28/40) and 50% (25/50), in Fujian, Guangdong, Henan, Hubei, Jiangshu, Shandong, Shanxi and Zhejiang respectively. In contrast, only six of 55 (11%) pet faecal samples (10 cats and 45 dogs) collected from three pet hospitals in Shenzhen were found to contain *mcr-1* (**Table 1**). A total of 590 strains containing the *mcr-1* element were isolated from animal faecal samples, with the majority being *E. coli *(n = 576), followed by *K. pneumoniae* (n = 10) and *Enterobacter cloacae* (n = 4) (**Table 1**).

#### Human faecal samples

Among the 150 human faecal samples tested, 85 were collected from inpatients and 65 were collected from healthy individuals who were admitted for physical examination. Twenty-two of the 85 faecal samples (26%) from inpatients and 20 of 65 samples (31%) from healthy individuals were found to contain strains that harboured the *mcr-1* gene. The majority of the *mcr-1*-bearing bacterial strains were confirmed to be *E. coli* (n = 84) (**Table 1**).

#### Water samples

Among the 24 sewage samples tested, six samples collected from the primary sedimentation tanks were negative, but all other 18 samples collected from other stages of water treatment were positive for *mcr-1*-bearing bacteria including water to be released to the sea after treatment (**Table 1**). The *mcr-1* gene was also detected in all six seawater samples collected from different locations in Shenzhen, but not in the four fresh water samples collected from the Meilin fresh water reservoir, a major source of fresh water in Shenzhen (**Table 1**). Among the water samples, *mcr-1*-bearing *E. coli* was the only bacterial species isolated from seawater; in contrast, bacterial species such as *K. pneumoniae* and *K. variicola* were isolated from sewage, although *E. coli* remained the dominant species (**Table 1**).

### Antimicrobial susceptibility profiles of *mcr-1*-bearing Enterobacteriaceae strains recovered from various sources

Randomly selected *mcr-1*-positive bacterial strains collected from various sources, as shown in **Table 2**, were subjected to assessment of their susceptibility to 16 antibiotics. Almost all of these strains exhibited a MIC of ≥ 4 µg/mL for colistin. Yet these colistin resistant strains were found to exhibit a diverse range of antibiotic susceptibility profiles, with co-resistance to antibiotics being a common phenomenon. 

**Table 2:** Antimicrobial susceptibility of *mcr-1*-bearing Enterobacteriaceae strains isolated from different sources, China^a^, December 2015−May 2016

Antibiotics	Resistance rate (%)					
	*Escherichia coli*					Non-*E. coli*
	Animal (n = 400)	Non-imported food (n = 400)	Overseas food (n = 100)	Human (84)	Water (n = 50)	(n = 34)
**AMC**	**22**	**3**	**0**	**0**	**0**	**21**
AMP	66	77	50	44	76	59
CRO	41	22	9	9	7	21
CTX	41	22	9	9	7	21
MRP	0	0	0	0	0	0
CIP	25	33	21	68	24	29
NAL	53	61	29	85	86	35
CLS	99	99	100	100	100	100
AMK	6	5	0	0	0	0
STR	72	53	59	29	17	29
CHL	49	73	38	0	69	41
KAN	63	55	21	12	76	41
TET	94	80	58	56	76	21
SXT	81	79	48	19	79	21
TIG	0	0	0	0	0	0
FOS	13	7	0	8	15	59

AMC: amoxicillin–clavulanic acid; AMK: amikacin; AMP: ampicillin; CHL: chloramphenicol; CIP: ciprofloxacin; CLS: colistin; CRO: ceftriaxone; CTX: ceftazidime; FOS: fosfomycin; KAN: kanamycin; MRP: meropenem; NAL: nalidixic acid; STR: streptomycin; SXT: trimethoprim–sulfamethoxazole; TET: tetracycline; TIG: tigecycline.

^a^ Some strains isolated from foods imported to China from overseas were also tested, as indicated inside the Table.

For example, up to 68% of colistin-resistant *E. coli* strains isolated from human faecal samples exhibited co-resistance to ciprofloxacin. Interestingly, organisms recovered from different sources also exhibited differential resistance profiles. In particular, *E. coli* strains recovered from food and animal faecal samples exhibited a higher rate of resistance to cephalosporins than those obtained from other sources (**Table 2**). A total of 257 cephalosporin-resistant *E. coli* isolates were obtained, from which 100 isolates were randomly selected and subjected to screening for the presence of different beta-lactamases. Ninety-three of the 100 isolates carried different types of *bla*_CTX-M_ genes, among which 75 belonged to *bla*_CTX-M-1_ group genes and the 18 remaining belonged to *bla*_CTX-M-9_ group genes (data not shown). For non-*E. coli* isolates, they were mainly *K. pneumoniae* isolates and showed a high resistance rate to cephalosporins and fosfomycin. Our data also showed that resistance to meropenem, amikacin and tigecycline remained extremely rare among *mcr-1*-positive strains, including those isolated from animals. Nevertheless, it should be noted that two *mcr-1* positive *E. coli* strains that exhibited cross-resistance to meropenem and colistin, as well as most other tested antimicrobial drugs, had been recovered from animal faecal products (data not shown).

### Genetic features of *mcr-1*

To investigate the genetic features of the *mcr-1* gene harboured by strains of different bacterial species, randomly selected *E. coli* strains (maximum one from each sample) isolated from various sources, as shown in **Table 3**, were subjected to assessment of their ability to undergo conjugative transfer of the *mcr-1* gene to *E. coli* J53.

**Table 3:** Sizes and conjugation rate of plasmids harbouring the *mcr-1* gene

Bacterial species / source of isolation		Number of isolates	Successful conjugation		Conjugation rate (%)	Unsuccessful conjugation (plasmid/chromosome)		
			Number	Approximate plasmid size in kb (number of isolates harbouring this plasmid)		Number	Approximate plasmid size range in kb (Number of isolates harbouring this plasmid)	Number on chromosome
*Escherichia coli*	Animal faeces	60	31	60 (14), 33 (17)	52	29	78−480 (19)	10
	Food	70	27	60 (14), 33 (13)	39	43	78−480 (31)	12
	Water	20	6	60 (3), 33 (3)	30	14	120−250 (12)	2
	Human faeces	50	38	60 (20), 33 (18)	76	12	78−480 (10)	2
Non-*E. coli* strains		34	21	60 (8), 33 (13)	62	38	78−250 (13)	0

**Figure 1:**
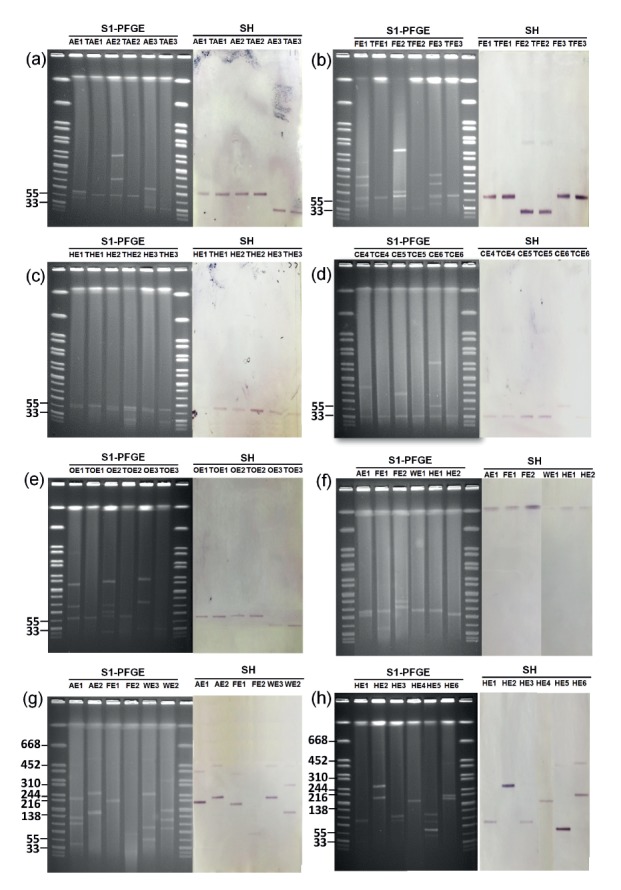
S1-PFGE and Southern hybridisation (SH) analysis of *mcr-1*-bearing conjugative and non-conjugative plasmids harboured by strains of *E. coli* or other Enterobacteriaceae species isolated from various sources, December 2015−May 2016

The transfer rate among *E. coli* strains isolated from different sources was significantly different (p = 0.0002) with *E. coli* strains obtained from human faeces and animals being the highest (**Table 3**). S1-PFGE characterisation and Southern hybridisation were then performed on transconjugants as well as on their parental *E. coli* strains, using *mcr-1*-specific probes, to determine the range of transmissible and non-transmissible *mcr-1*-positive elements harboured by the test strains. Two major conjugative plasmids which contained the *mcr-1* gene, with a size of ca 33 kb or 60 kb respectively, were detectable in most of the *E. coli* strains recovered from various sources and their corresponding transconjugants. On the other hand, the size of non-conjugative *mcr-1*-bearing plasmids in *E. coli* isolated from various sources varied (ca 78 kb − ca 480 kb). Interestingly, some *E. coli* strains, mostly in animal products, were found to harbour a chromosomal *mcr-1* gene (**Figure 1** **,** **Table 3**). Apart from *E. coli*, 21 of 34 non-*E. coli* strains were found to successfully transfer their colistin resistance phenotypes to the recipient strains through the ca 33 kb or 60 kb plasmids, whereas the sizes of non-conjugative plasmids ranged from ca 78 kb − ca 250 kb (**Figure 1** **,** **Table 3**).

PFGE: pulsed-field gel electrophoresis.

(a) Representative *E. coli* strains recovered from animal faeces (AE), transconjugants which have acquired plasmids recovered from animal *E. coli* isolates (TAE); (b) Representative *E. coli* strains recovered from food specimens (FE), transconjugants which have acquired plasmids recovered from food-borne *E. coli* strains (TFE); (c) Representative *E. coli* strains recovered from human faeces (HE), transconjugants which have acquired plasmids recovered from human faecal *E. coli* isolates (THE); (d) Representative *E. coli* strains recovered from human clinical specimens (CE), transconjugants which have acquired plasmids recovered from human clinical *E. coli* isolates (TCE); (e) Other Enterobacteriaceae species recovered from various sources (OE), transconjugants which have acquired plasmids recovered from strains of other Enterobacteriaceae species (TOE); (f) *E. coli* from various sources carrying *mcr-1* on the chromosome; *E. coli* strains recovered from water samples (WE); (g,h) Representative non-conjugative plasmids recovered from *E. coli* in animal/ food / water and human faecal specimens.

### Genetic features of the most common transmissible *mcr-1*-positive plasmids

**Figure 2:**
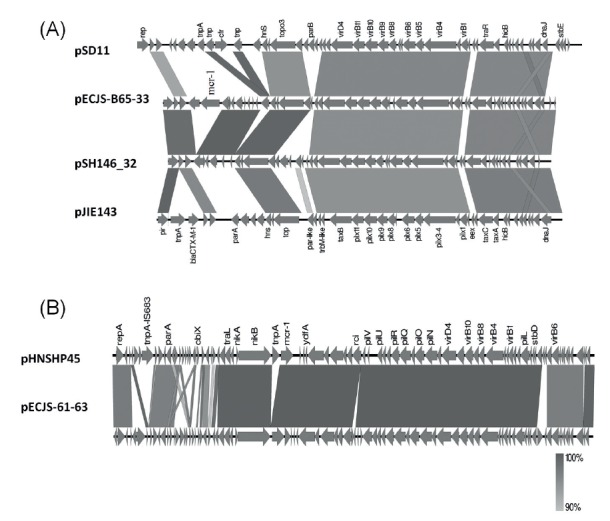
Comparison of the ca 33 kb and ca 60 kb *mcr-1*-bearing conjugative plasmids found in this study with other previously reported plasmids

One representative ca 33 kb conjugative plasmid (pECJS-B65–33) was sequenced and shown to belong to the IncX4 type, with a size of 33,298 bp and 41.85% GC content (**Figure 2**). Several similar plasmids have been reported from various parts of the world. One representative ca 60 kb plasmid (pECJS-61–63) was also sequenced and shown to belong to the IncI2 type, with a size of 63,656 bp and 42.64% GC content. It shares 99% similarity and 88% coverage with the pHNSHP45 plasmid except that *ISApI1* is missing in the upstream region of in *mcr-1* (**Figure 2**) [5].

(A) Comparison of pECJS-B65–33 with other IncX4 plasmids with similar backbone structure (plasmid name and the corresponding GenBank accession number): pSD11(KM212169) carrying *cfr* was isolated from an animal *E. coli* strain in China; pSH146_32(JX258655), which was known to contain a backbone highly similar to pECJS-B65–33 but without any antimicrobial resistance gene, was isolated from a *Salmonella Heidelberg* strain in the United States; pJIE143(JN194214) carrying *bla*_CTX-M-15_ was recovered from a clinical *E. coli* ST131 strain isolated in Sydney, Australia. The gene labels follow that of the original annotations in GenBank. Gene alignment was performed by EasyFigure 2.1 [22]. (B) Genetic comparison of pECJS-61–63 with pHNSHP45(KP347127), the first *mcr-1*-bearing IncI2 plasmid reported in the literature. The upstream region of *mcr-1* in pECJS-61–63 was found to lack *ISApI1* when compared with pHNSHP45.

## Discussion

Just a few months after the discovery of the plasmid-mediated colistin resistance gene, *mcr-1*, a flood of information regarding this gene was reported in the literature [5,14-17]. However, these studies failed to provide comprehensive understanding on the distribution of *mcr-1*-like elements in various ecological niches in order to assess the potential impact of dissemination of this novel colistin resistance element on current antimicrobial treatment protocols, especially the use of colistin to treat infections due to CRE.

In this study, we developed a sensitive and specific method for the isolation of *mcr-1*-bearing bacteria from various sources and used it to investigate the prevalence of *mcr-1* in various sample types collected from different settings. It should be noted that the traditional selective isolation method using colistin-containing agar plates is not suited for isolation of *mcr-1*-positive bacteria due to the prevalence of organisms that are intrinsically resistant to colisin, such as the *Proteus*, *Morganella*, *Neisseria*, *Providencia* and *Serratia* spp., some of which are normal flora of animals and human, rendering measurement of the true *mcr-1* positive rate among Enterobacteriaceae species challenging. In this work, we tried to focus on isolation of Enterobacteriaceae strains harbouring the *mcr-1* gene. To eliminate non-Enterobacteriaceae strains before plating, we added another selective step by diluting peptone water enrichment broth in MEE broth (selective for Enterobacteriaceae) supplemented with 2 µg/mL colistin, which was proven to be optimal in selecting strains harbouring *mcr-1*. Using this in-house method, we were able to isolate different species of *mcr-1*-positive bacteria including Enterobacteriaceae and other species exhibiting intrinsic resistance to colistin. During the course of this study, two new variant of the plasmid-mediated colistin resistance gene, namely *mcr-2* and *mcr-3* were also discovered [18,19]. We screened all isolates that were positive for *mcr-1* for the presence of these two variants, but none of these isolates contained any of these two variants (data not shown). 

It became difficult to determine the origin of *mcr-1* since it has been disseminated to various species of bacteria. However, comprehensive data generated by this study suggested that the *mcr-1* gene may originate from *E. coli* in animal gastrointestinal (GI) tract due to prolonged usage of colistin in livestock. Pet animals which are rarely exposed to colistin exhibited a much lower level of prevalence of *mcr-1*-positive organisms than pigs and chickens. Additional evidence supporting this hypothesis includes findings that *E. coli* is the predominant species among *mcr-1*-bearing Enterobacteriaceae strains and that *mcr-1* was detectable in *E. coli* isolated from animals during the 1980s [20], a date much earlier than that of the first detection of this gene in human (2008) in a retrospective study [14]. Enterobacteriaceae strains carrying *mcr-1* in the animal GI tract can cause contamination of their meat products and the environment as evidenced by the highly prevalent *mcr-1*- positive Enterobacteriaceae strains in food, waste water and seawater, but not in a fresh water reservoir that is not contaminated by faeces. Findings of this work also confirmed that *mcr-1* is an extremely common mobile element detectable worldwide, and commonly recoverable from food products originating from different parts of the world, including Australia, the most geographically isolated continent. 

One limitation in investigating food samples from overseas in our study was however the uncertainty in the sources of contamination. The food products could have been contaminated in the country of origin or during the re-packaging process after they were imported into Hong Kong. Another limitation of the current study is that it involved non-probability sampling. Hence, while many samples were investigated, and new information on *mcr-*1-bearing Enterobacteriaceae in various ecological niches was provided, the extent of data representativeness of the areas under study is difficult to derive.

**Figure 3:**
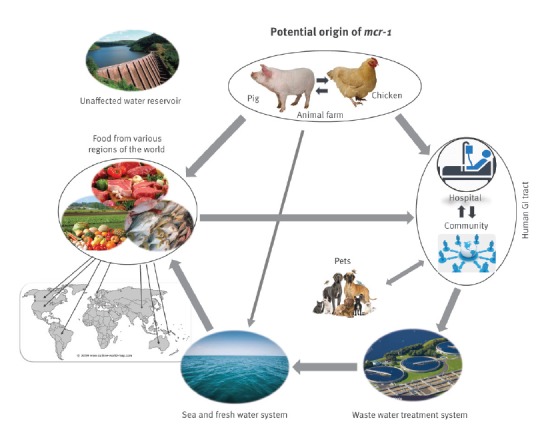
Potential transmission route of *mcr-1* in the ecosystem

Based on the molecular epidemiology data in this study, we propose a potential *mcr-1* transmission route. The *mcr-1* gene may have evolved from animal GI tract with the prolonged use of colistin as a growth promoter in livestock. The *mcr-1* gene might have then been transmitted to humans through the food chain or direct contact between animals and humans, as well as through contamination of the fresh and seawater system, which in turn lead to contamination of vegetables and seafood. The persistence of *mcr-1* in the human GI tract microflora can cause further contamination of our water systems through improper disposal of waste water. A fresh water reservoir that is outside these transmission routes maintained clear of *mcr-1* contamination (**Figure 3**).

The *mcr-1* gene may undergo evolutionary changes in the animal GI tract upon prolonged usage of colistin as growth promoter. The gene is then transmitted to human through the food chain or direct human contact with animals, as well as through contamination of the fresh and seawater system, which in turn cause contamination of vegetables and seafood. The persistence of *mcr-1* in the human GI tract microflora can cause further contamination of the water systems through disposal of waste water containing human faeces. Fresh water systems outside these transmission routes remain clear of *mcr-1* contamination.

Genetic characterisation of *mcr-1*-bearing plasmids revealed that the gene may reside in both chromosome and plasmids, but most commonly on two conjugative elements of ca 33 and 60 kb in size. A much higher proportion of Enterobacteriaceae strains in the human GI tract and clinical specimens was found to carry these two conjugative plasmids when compared with Enterobacteriaceae strains recovered from other sources, suggesting that the prevalence of *mcr-1* among human Enterobacteriaceae strains is mainly due to the transmission of *mcr-1*-bearing conjugative plasmids to the human GI tract microflora. The fact that *mcr-1*-bearing organisms recoverable from human faecal samples and clinical specimens exhibit highly different antibiotic susceptibility and PFGE profiles (data not shown) from those of other sources also supports the idea that plasmids may play an important role in *mcr-1* transmission to humans. Sequence analysis revealed the presence of *mcr-1*-negative plasmids with a backbone similar to that of the 33 and 60 kb elements in Enterobacteriaceae strains isolated from animals, thus further supporting the theory that these two conjugative plasmids originated from animals [16]. The finding that these two plasmids are highly conjugative (conjugation efficiency at 10 ^− 1^ level) [21] and stably inherited in Enterobacteriaceae strains in the human GI tract without colistin selective pressure suggests that they may severely compromise efforts to control dissemination of the *mcr-1* among bacterial pathogens. It is apparently too late to eradicate organisms harbouring *mcr-1*. Upon approval of clinical use of colistin in China and other regions of the world this year or in the near future, the two colistin resistance-encoding plasmids described in our study may potentially spread in the hospital environment within a short period. The use of colistin to treat CRE infections may result in rapid selection of organisms that exhibit resistance to both carbapenems and colistin. This highlights the importance of vigilance and antimicrobial stewardship. Development of effective inhibitors for MCR-1 or intervention measures to disrupt the transmission of these two plasmids may be an effective strategy to prolong the use of colistin as a last-line antibiotic to treat life-threatening bacterial infections.
